# Production of an anti-angiogenic factor sFLT1 is suppressed via promoter hypermethylation of *FLT1* gene in choriocarcinoma cells

**DOI:** 10.1186/s12885-020-6598-9

**Published:** 2020-02-10

**Authors:** Tadashi Sasagawa, Atsushi Jinno-Oue, Takeshi Nagamatsu, Kazuki Morita, Tetsushi Tsuruga, Mayuyo Mori-Uchino, Tomoyuki Fujii, Masabumi Shibuya

**Affiliations:** 1 Institute of Physiology and Medicine, Jobu University, 270-1 Shin-machi, Takasaki, Gunma 370-1393 Japan; 2 Bioresource Center, Gunma University Graduate School of Medicine, 3-39-22 Showa-machi, Maebashi, Gunma 371-8511 Japan; 3 Department of Obstetrics and Gynecology, Graduate School of Medicine, The University of Tokyo, 7-3-1 Hongo Bunkyo-ku, Tokyo, 113-8655 Japan

**Keywords:** Choriocarcinoma, DNA methylation, sFLT1, Trophoblast, Tumor suppressor gene

## Abstract

**B**ackground**:**

Soluble Fms-like tyrosine kinase-1 (sFLT1) as an anti-angiogenic factor is abundantly expressed in placental trophoblasts. Choriocarcinoma, a malignant tumor derived from trophoblasts, is known to be highly angiogenic and metastatic. However, the molecular mechanism underlying angiogenesis in choriocarcinoma pathogenesis remains unclear. We aimed to investigate the mRNA expression and DNA methylation status of the *FLT1* gene in human choriocarcinoma cells and trophoblast cells.

**Methods:**

qRT-PCR, Western blotting and ELISA were conducted to evaluate the mRNA and protein expression levels of sFLT1. 5-aza-2′-deoxycytidine (5azadC) treatment and bisulfite sequencing were used to study the *FLT1* gene promoter methylation. The effect of sFLT1 on choriocarcinoma growth and angiogenesis was evaluated in a xenograft mouse model.

**Results:**

Expression of the *FLT1* gene was strongly suppressed in choriocarcinoma cell lines compared with that in the primary trophoblasts. Treatment of choriocarcinoma cell lines with 5azadC, a DNA methyltransferase inhibitor, markedly increased in mRNA expression of three *FLT1* splice variants and secretion of sFLT1 proteins. Bisulfite sequencing revealed that the CpG hypermethylation was observed at the *FLT1* promoter region in choriocarcinoma cell lines and a human primary choriocarcinoma tissue but not in human trophoblast cells. Interestingly, in 5azadC-treated choriocarcinoma cell lines, *sFLT1* mRNA expression and sFLT1 production were further elevated by hypoxic stimulation. Finally, as expected, sFLT1-expressing choriocarcinoma cells implanted into nude mice showed significantly slower tumor growth and reduced microvessel formation compared with GFP-expressing control choriocarcinoma cells.

**Conclusions:**

Inhibition of sFLT1 production by *FLT1* silencing occurs via the hypermethylation of its promoter in choriocarcinoma cells. The stable expression of sFLT1 in choriocarcinoma cells resulted in the suppression of tumor growth and tumor vascularization in vivo. We suggest that the *FLT1* gene may be a cell-type-specific tumor suppressor in choriocarcinoma cells.

## Background

Choriocarcinoma is a rare cancer characterized by highly pro-angiogenic and metastatic malignant epithelial tumors, which can be divided into gestational and non-gestational types based on differences in the onset of symptoms [1–4]. Gestational choriocarcinoma derives from molar pregnancies, non-molar abortions, and preterm or term deliveries. Non-gestational choriocarcinoma emerges from gonadal germ cell tumors in males or females, but can occur from other epithelial cancers such as lung, stomach, and bowel. Commonly, choriocarcinoma presents with elevated expression of chorionic gonadotropin, and is histologically composed of cytotrophoblasts, intermediate trophoblasts or syncytiotrophoblasts [1]. Although both types of choriocarcinoma are pathologically similar, it has been shown that their genetic origin, immunogenicity, sensitivity to chemotherapy, and prognosis differ [3]. However, the mechanisms underlying the development and pathogenesis of choriocarcinoma remain to be elucidated.

Pro-angiogenic factors such as vascular endothelial growth factor (VEGF) and placental growth factor (PlGF) are known to play important roles in angiogenesis and vasculogenesis. Soluble Fms-like tyrosine kinase-1 (sFLT1) is a truncated form of the transmembrane tyrosine kinase receptor FLT1 (VEGF receptor-1) that tightly binds VEGF and PlGF [5]. sFLT is generated by the alternative splicing and premature termination of *FLT1* pre-mRNA, retaining the 1 to 6 immunoglobulin domains of the FLT1 extracellular ligand-binding region [6–8]. It is known to function as a decoy, sequestering VEGF and preventing the initiation of intracellular signal transduction. sFLT1 exists as only one isoform in mice and chickens [9, 10], whereas four sFLT1 isoforms have been reported so far in humans [7, 11–13]. Among these, sFLT1-i13 and sFLT1-e15a are observed abundantly in the human body. Notably, the former is expressed in various types of cells while the latter is predominantly expressed in the placenta [14]. Moreover, in placental tissues in situ hybridization has revealed that most of the *sFLT1-i13* and *sFLT1-e15a* mRNA is localized within trophoblasts, which are fetal cells located between the fetal and maternal blood vessels [14, 15]. It is suggested that in the placenta, trophoblast-derived sFLT1 maintains the physiological vascular integrity of the placental tissue by sequestering excess VEGF produced in response to mild hypoxia. Abnormal sFLT1 production by trophoblasts induces the development and progression of preeclampsia by antagonizing the activity of VEGF and PlGF, leading to maternal endothelial dysfunction, which causes hypertension and proteinuria [16].

The inactivation of tumor suppressor genes by gene silencing, due to epigenetic alterations, gene mutations, or deletions, is known to contribute to the development and progression of cancer [17]. One gene silencing mechanism involves the abnormal methylation of promoter CpG sites by methyltransferases. Indeed, in choriocarcinoma it has been reported that DNA hypermethylation occurs not only in tumor-suppressor genes, but also in extracellular matrix remodeling genes and stem cell transcription factors [18, 19]. Although sFLT1 is abundantly expressed in trophoblasts, choriocarcinomas are shown to be highly pro-angiogenic, therefore we hypothesized that sFLT1 production is inhibited by epigenetic alterations in choriocarcinoma. In this study, the mRNA expression and DNA methylation status of the *FLT1* gene were investigated in human primary trophoblasts, human choriocarcinoma cell lines (BeWo, JAR, and JEG-3) and primary choriocarcinoma tissue. We found that sFLT1 production is inhibited by *FLT1* gene silencing via hypermethylation of its promoter in choriocarcinoma cell lines and primary choriocarcinoma tissue.

## Methods

### Cell lines and culture

BeWo (Japanese Collection of Research Bioresources (JCRB) Cell Bank, Tokyo, Japan; JCRB9111), JAR (American Tissue Culture Collection (ATCC), Manassas, VA, USA; HTB-144), and JEG-3 (ATCC; HTB-36) choriocarcinoma cell lines were maintained in Ham’s F-12 medium (Nacalai Tesque, Inc., Kyoto, Japan) containing 10% fetal bovine serum (FBS), 100 U/mL penicillin, and 100 μg/mL streptomycin. HTR-8/SVneo cells, which are human first-trimester trophoblasts immortalized with the Simian virus 40 large T antigen, and HEK293 cells were kindly provided by Dr. Charles Graham (Queen’s University, Kingston, Canada) and Prof. Hiroto Shimojo (University of Tokyo, Tokyo, Japan), respectively. HEK293 cells and HTR-8/SVneo cells were cultured in Dulbecco’s modified Eagle’s medium (DMEM; Nacalai Tesque, Inc.) or a 1:1 mixture of DMEM/Ham’s F-12, respectively, supplemented with 10% FBS and antibiotics. All cells were cultured at 37 °C in a humidified atmosphere with 5% CO_2_.

To induce DNA demethylation, choriocarcinoma cells were treated with either 5-aza-2′-deoxycytidine (5azadC; Sigma-Aldrich, St. Louis, MO, USA) at different doses (from 1 to 50 μM) for 3 days or with 10 μM 5azadC for 5 days. Dimethyl sulfoxide (DMSO; Sigma-Aldrich) was used as a control vehicle. Culture media were changed daily to maintain the stability of 5azadC during treatment.

For the hypoxia experiments, choriocarcinoma cells were subjected to 5azadC treatment for 5 days. After changing to fresh medium, the cells were exposed to 2% O_2_, 5% CO_2_, and 93% N_2_ for 24 h in a MultiGas incubator (WAKEN 9000EX; WAKEN B TECH Co., Ltd., Kyoto, Japan).

### Isolation and culture of human villous cytotrophoblasts

After obtaining informed consent, normal term placentas were collected from healthy pregnant women at 37 to 40 weeks of gestation at cesarean delivery due to breech presentation and previous cesarean section. Human villous cytotrophoblasts were isolated from the villous tissues of the placentas as described previously [20]. Purified cytotrophoblasts were cryopreserved at − 80 °C in a Cellbanker (ZENOAQ, Fukushina, Japan) until further use.

Thawed cytotrophoblasts were suspended in a 1:1 mixture of DMEM/Ham’s F-12 containing 10% FBS and antibiotics. The cells were seeded into type I collagen-coated culture plates or dishes (Sumilon Celltight; Sumitomo Bakelite Co., Ltd., Tokyo, Japan) at a density of approximately 2.3 × 10^5^ cells/cm^2^ and then incubated for 24 h (cytotrophoblast preparation). After replacement of the culture media, the cytotrophoblasts were incubated for an additional 48 h for syncytium formation (syncytiotrophoblast preparation), and conditioned media were collected for Western blotting.

### Clinical specimen

After obtaining informed consent, a choriocarcinoma tissue was obtained from a cancer patient at the University of Tokyo Hospital. The collected tissue was stored at − 80 °C for qRT-PCR and Bisulfite sequencing.

### Quantitative real-time polymerase chain reaction (qRT-PCR)

RNA was extracted and mRNA expression was assessed using qRT-PCR as described previously [21]. All data were normalized to *β-actin* expression or *GAPDH* expression. Each value was obtained from the mean of three independent experiments. The oligonucleotide primer sequences are listed in Additional file 1: Table S1.

### Western blotting

Cell lysates were prepared with lysis buffer (50 mM Tris-HCl pH 8.0, 150 mM NaCl, and 1% Triton X-100) supplemented with a protease inhibitor cocktail (Nacalai Tesque, Inc.). Protein concentration was determined using a protein assay kit (Bio-Rad, Hercules, CA, USA). Proteins were separated and transferred to polyvinylidene fluoride membranes by gel electrophoresis and electroblotting, respectively. The following primary antibodies were used: anti-human FLT1 N-terminal region (1:1000) [22] and anti-β-actin (1:500; Cell Signaling Technology, Beverly, MA, USA). Bands were visualized using an ECL Western Blotting Detection System (GE Healthcare, Uppsala, Sweden) on a chemiluminescence imaging system (KETA C Plus; Wealtec Corp., Sparks, NV, USA).

### Immunoprecipitation

The cell lysates were subjected to immunoprecipitation using Dynabeads Protein G (Invitrogen) bound to anti-human FLT1 monoclonal antibody reacted with the first Ig-like domain of FLT1 (KM1730) as described previously [21]. The precipitated proteins were subjected to Western blotting as described above.

### Heparin-affinity pull-down for concentrating sFLT1 proteins

Secreted sFLT1 isoforms within the conditioned medium were concentrated using a previously described pull-down method with Heparin-Sepharose beads [21].

### Bisulfite sequencing

Genomic DNA was extracted from uncultured cytotrophoblasts, cultured cell lines and a tumor specimen using a NucleoSpin Tissue kit (Macherey-Nagel, Düren, Germany). The bisulfite modification procedure was carried out using a MethylEasy Xceed Rapid DNA Bisulphite Modification Kit (Human Genetic Signatures Pty, Randwick, Australia) according to the manufacturer’s instructions. Amplification was performed using EpiTaq HS (Takara Bio) and PCR primers for the detection of 5′ region in *FLT1* gene (forward 5′-GTAGGAGGAGGGGTAAGGGTAA-3′ and reverse 5′-ACTCCAACCAAAAAACAACCA-3′). These primers were designed using Methyl Primer Express Software v1.0 (Applied Biosystems Inc. Foster City, CA, USA). The thermal cycling conditions consisted of an initial activation cycle (98 °C for 20 s), followed by 40 cycles of denaturation (98 °C for 10 s), annealing (55 °C for 30 s), and amplification (72 °C for 30 s). The PCR products were then cloned into pGEM-T easy vectors (Promega), and DNA sequencing was performed on 20 clones from each sample.

### Enzyme-linked immunosorbent assay (ELISA)

The concentration of sFLT1 and VEGF-A in the conditioned media was quantitatively measured by ELISA kits which are commercially available (R&D Systems Inc., Minneapolis, MN, USA). To normalize the sFLT1 and VEGF-A secretion volumes according to cell number, the number of cells was counted using a hemocytometer or estimated from the measured cell lysate protein content.

### Establishment of sFLT1-i13- or GFP-expressing JEG-3 cells

To construct expression vectors for sFLT1-i13 or green fluorescent protein (GFP), DNA fragments encoding these molecules were digested using pVL-6 N-Flt [22] and pEGFP-N1 (Clontech, Mountain View, CA, USA), respectively, then cloned into a bovine papilloma virus-based plasmid vector pBCMGSneo [23]. To establish stable sFLT1-i13- or GFP-expressing JEG-3 cells, each vector was transfected into JEG-3 cells using Lipofectamine 2000 (Invitrogen) according to the manufacturer’s instructions. Cells were selected in the presence of 600 μg/mL G418 (Nacalai Tesque, Inc.) and then maintained in a growth medium containing 200 μg/mL G418. These cells were designated as JEG3-i13 and JEG3-GFP, respectively. Cell proliferation assay is described in details in Additional file 2.

### In vivo tumorigenicity assay

All animals were bred at the Bioresource Center of Gunma University. BALB/c-nu/nu female nude mice (Charles River Laboratories Japan, Inc., Kanagawa, Japan) were maintained in cages (4–5 animals per cage) under a 12 dark/light cycle at a constant temperature of 23 ± 1 °C with free access to food and water during all the experiment. JEG-3 cells expressing sFLT1-i13 or GFP (6 × 10^6^ cells) were resuspended in 200 μL of Ham’s F-12 medium without FBS and antibiotics, then injected into the subcutaneous space of 6-week-old mice anesthetized with inhaled isoflurane. Total nine mice, 5 for JEG3-sFLT1-i13 cells and 4 for JEG3-GFP cells, were used to obtain statistically significant results. Tumor size was measured every third day using a digital vernier caliper and their volume was calculated according to the following formula: (length × width^2^) × 0.5.

### Quantitative analysis of microvessel density

Fifteen days after transplantation, xenograft mice were euthanized by cervical dislocation to obtain tumor tissues. After the treatment, animal death was confirmed. The tissues were fixed with 4% paraformaldehyde solution (Muto Pure Chemicals, Tokyo, Japan). The specimens were embedded in paraffin and sectioned. For immunostaining of the host microvessels, specimens were treated with a 1:250 dilution of rat anti-mouse CD31 monoclonal antibody (clone SZ31) (Dianova GmbH, Hamburg, Germany), and visualized using a Histofine Simple Stain Mouse MAX PO detection kit (Nichirei Bioscience, Tokyo, Japan). The nuclei were counterstained with hematoxylin. Images of the tumor sections were obtained using a microscope (IX70; Olympus, Tokyo, Japan) equipped with a C5810 color chilled 3CCD camera system (Hamamatsu Photonics, Shizuoka, Japan). Microvessels were defined as any CD31 positive endothelial cell or endothelial cell cluster with or without a definable lumen. The immunostained tumor sections were prepared from each implanted mouse at five sites and the number of microvessels was counted in independently three times per tumor section in a blinded fashion using a 20 × objective lens. The results were expressed as the average number of microvessels per mm^2^ area.

### Statistical analysis

Data are expressed as the mean ± standard deviation (SD) and parametric data were analyzed using an unpaired *t*-test. Statistical analyses were performed using Excel 2011 (Microsoft, Seattle, WA, USA) with the Statcel4 (OMS, Tokyo, Japan) “add-in” software. A *P* value < 0.05 was considered statistically significant.

## Results

### Up-regulation of *VEGF-A* mRNA expression in choriocarcinoma cells

**Fig. 1:**
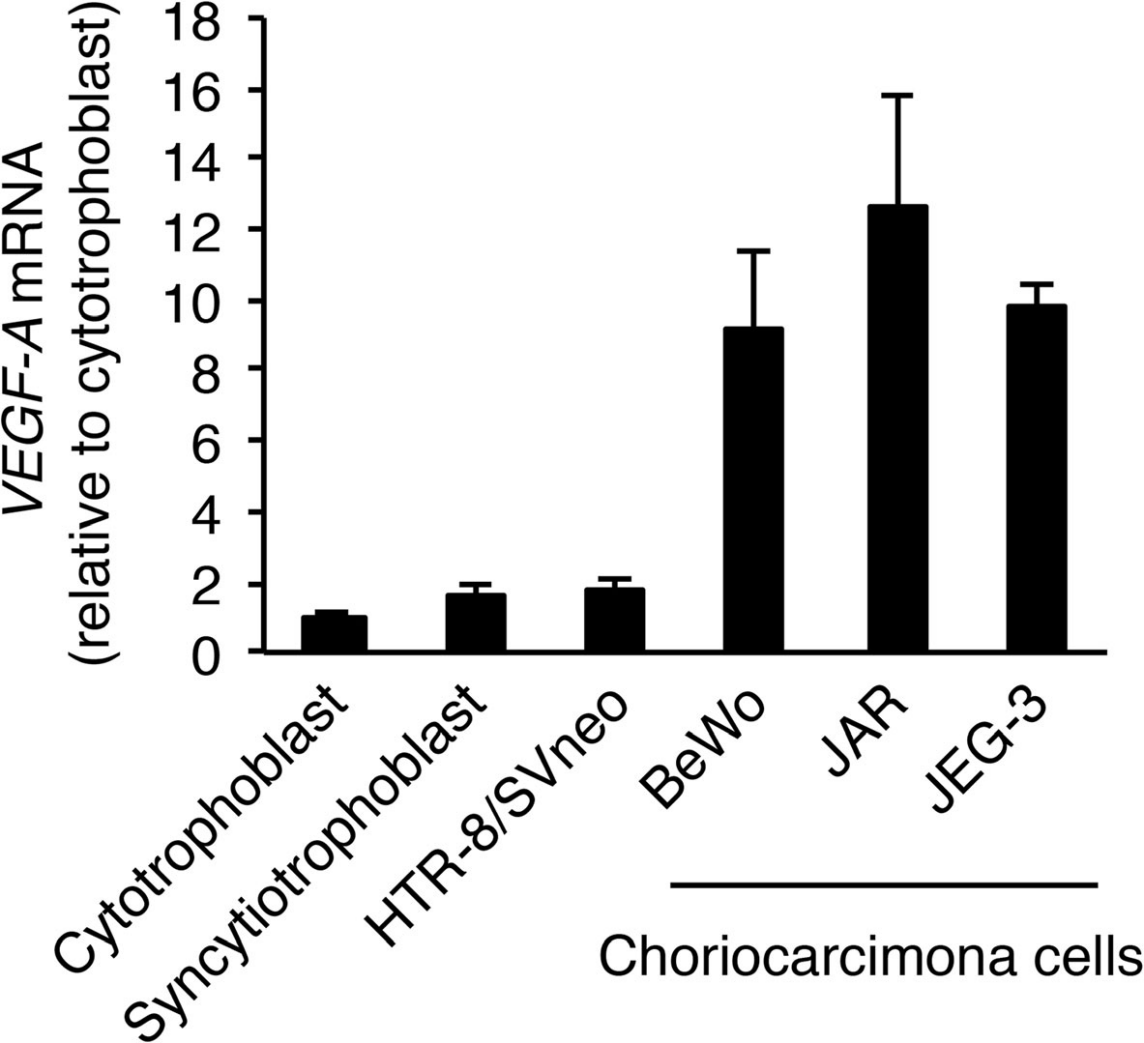
*VEGF-A* mRNA expression is up-regulated in choriocarcinoma cells. Cytotrophoblasts (cultured for 24 h after seeding) and syncytiotrophoblasts (obtained by spontaneous differentiation of the cytotrophoblasts after 72 h culture) were prepared. *VEGF-A* expression was measured by qRT-PCR using *β-actin* mRNA as a reference. Results are expressed as a fold change relative to cytotrophoblasts. All values represent the mean ± SD (*n* = 3)

Choriocarcinoma, a malignant trophoblastic cancer, is known to be highly pro-angiogenic [4]. It has previously been reported that the protein expression level of VEGF, a pro-angiogenic factor, is higher in choriocarcinoma cell lines than in normal trophoblastic cell lines [22]. Therefore, we first measured the mRNA expression level of *VEGF-A* in human primary trophoblasts, immortalized human trophoblasts (HTR-8/SVneo), and choriocarcinoma cell lines (BeWo, JAR and JEG-3) by qRT-PCR analysis. Human primary cytotrophoblasts have been shown to differentiate into syncytiotrophoblasts via spontaneous cell fusion [23]. Correspondingly, after 72 h under our culture conditions the morphological change into multinuclear cell clusters was observed (data not shown). In this study, cytotrophoblasts were cultured for 24 h after seeding, and syncytiotrophoblasts were obtained by spontaneous differentiation of cytotrophoblasts after 72 h of culture. *VEGF-A* mRNA expression was significantly increased in choriocarcinoma cells compared with that in human trophoblast cells (Fig. 1), suggesting that *VEGF-A* expression is up-regulated when trophoblast cells transform into malignant cells.

### Inhibition of sFLT1 production in choriocarcinoma cells

We were the first to report that three *FLT1* transcripts are expressed in human embryo kidney 293 (HEK293) cells [6]. We confirmed the Northern blot analysis finding using qRT-PCR and Western blotting. The mRNA and protein expression of transmembrane FLT1 (tmFLT1), sFLT1-i13, and sFLT1-e15a was observed in HEK293 cells (Additional file 1: Figure S1). Therefore, in this study, cell lysates and conditioned media from HEK293 cells were used as a positive control for FLT1 isoforms in Western blot analysis.

**Fig. 2:**
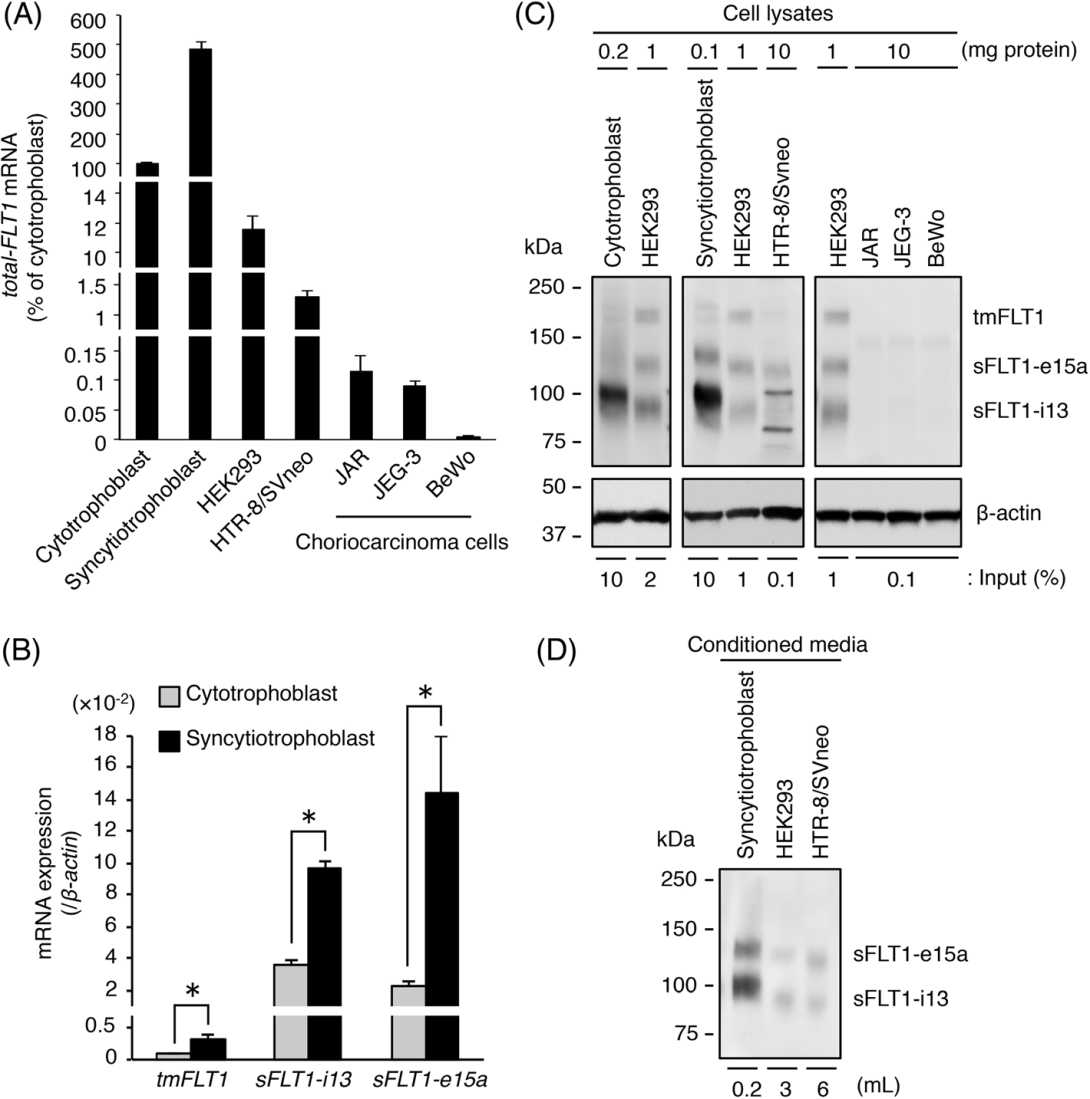
Expression of three FLT1 isoforms is inhibited in choriocarcinoma cells. **a** The mRNA expression levels of all *FLT1* transcript variants (*total-FLT1*) in several cells were measured by qRT-PCR using *β-actin* mRNA as a reference. Results are expressed as a percentage relative to cytotrophoblasts. **b** The mRNA expression levels of three *FLT1* splice variants in cytotrophoblasts and syncytiotrophoblasts. Results are expressed as a ratio relative to the expression of *β-actin* mRNA. **c** Immunoprecipitation of FLT1 isoforms from the cell lysates. The immunoprecipitates and cell lysates (input) were subjected to Western blotting. β-actin was used as a loading control. Numbers show the amount of protein subjected to immunoprecipitation. The indicated percentage of cell lysate was used as the input. **d** Western blotting of sFLT1 proteins secreted into conditioned media. Numbers show the volume of media subjected to heparin-affinity pull-down. Uncropped images of Western blots are presented in Additional file 1: Figure S6. All values represent the mean ± SD (*n* = 3)

Although sFLT1 is abundantly expressed in placental trophoblasts, choriocarcinomas have high pro-angiogenic activity, therefore we hypothesized that sFLT1 production is inhibited in choriocarcinoma cells. *FLT1* expression was compared in human trophoblast and choriocarcinoma cells using qRT-PCR with primers covering all *FLT1* transcript variants, including *tmFLT1* and *sFLT1* mRNAs. The *tmFLT1* plus *sFLT1s* amplicon was designated as *total-FLT1*. In primary trophoblasts, the *total-FLT1* mRNA level increased by approximately 5-fold when cytotrophoblasts differentiated into syncytiotrophoblasts (Fig. 2a). Moreover, the mRNA expression levels of *tmFLT1*, *sFLT1-i13*, and *sFLT1-e15a* were significantly up-regulated (Fig. 2b). The mRNA level of *total-FLT1* in HTR-8/SVneo cells was approximately one-hundredth of that in cytotrophoblasts (Fig. 2a), and it was strongly down-regulated in the three choriocarcinoma cell lines compared with that in cytotrophoblasts (Fig. 2a). Interestingly, the *total-FLT1* mRNA expression level was very low in a human primary choriocarcinoma tissue specimen compared with that in the three choriocarcinoma cell lines (Additional file 1: Figure S2).

Western blot analysis of the immunoprecipitated cell lysates showed that three FLT1 isoforms were detected in human trophoblast cells (Fig. 2c), but not in the three choriocarcinoma cell lines (Fig. 2c). As expected, sFLT1 e15a and sFLT1-i13 were also observed in the conditioned media from human trophoblast cells (Fig. 2d), but not the three choriocarcinoma cell lines (data not shown). These results indicate that the production and secretion of sFLT1 proteins were very low in the three choriocarcinoma cells.

### DNA methyltransferase inhibitor enhances the mRNA expression of three *FLT1* splice variants and induces sFLT1 production in choriocarcinoma cells

Since sFLT1 proteins were not secreted at detectable levels in three choriocarcinoma cell lines, we hypothesized that CpG sites located in the promoter region of *FLT1* gene are methylated. To determine whether the inhibition of sFLT1 production resulted from promoter methylation, the three choriocarcinoma cell lines were treated with the DNA methyltransferase inhibitor, 5-aza-2′-deoxycytidine (5azadC). We first evaluated the dose-dependent effects of 5azadC on *FLT1* gene expression in the three cell lines by exposing the cells to different concentrations of 5azadC for 3 days. As shown in Additional file 1: Figure S3A, the levels of *total-FLT1* mRNA expression increased in a dose-dependent manner, with a 5azadC concentration of 10 μM sufficient to achieve optimal induction levels. After treatment with 10 μM of 5azadC for 5 days, *total-FLT1* mRNA expression was up-regulated in all three cells (Additional file 1: Figure S3B). Thus, the effect of DNA demethylation by 5azadC was similar in all choriocarcinoma cell lines.

**Fig. 3:**
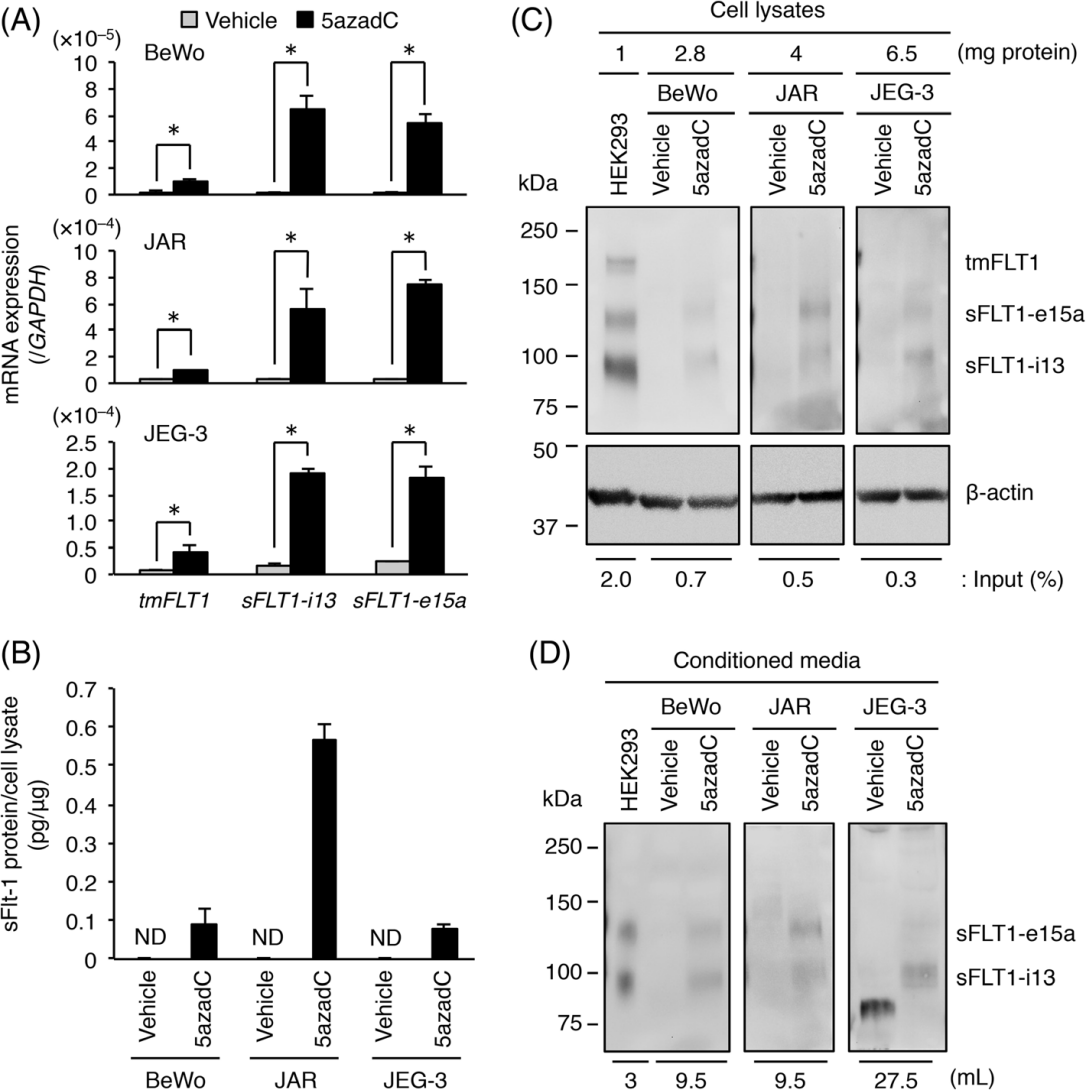
5-aza-2′-deoxycytidine (5azadC) enhances *FLT1* expression and induces sFLT1 secretion in choriocarcinoma cells. Three choriocarcinoma cell lines were incubated for 5 days in the presence of 0.1% DMSO (vehicle control) or 10 μM 5azadC. Culture media were changed daily and on the fifth day the conditioned media were collected for ELISA and Western blotting. **a** The mRNA expression levels of three *FLT1* splice variants in the three choriocarcinoma cell lines. Results are expressed as a ratio relative to the expression of *GAPDH* mRNA. **b** Measurement of sFLT1 secreted from the three choriocarcinoma cell lines. The amount of sFLT1 in the conditioned media was measured by ELISA, and was then normalized to the protein content of each cell. **c** Immunoprecipitation of FLT1 isoforms from the cell lysates. Numbers represent the amount of protein subjected to immunoprecipitation. The indicated percentage of cell lysate was used as the input. **d** Western blotting of sFLT1 proteins secreted into the conditioned media. Numbers show the volume of media subjected to heparin-affinity pull-down. Uncropped images of Western blots are presented in Additional file 1: Figure S6. All values represent the mean ± SD (n = 3). Asterisks indicate a significant difference (*P* < 0.05). ND: not detected

Next, we examined the mRNA expression levels of three *FLT1* splice variants in choriocarcinoma cells treated with 10 μM 5azadC for 5 days. Culture media were changed daily and on the fifth day the conditioned media were collected. As shown in Fig. 3a, the mRNA expression levels of *tmFLT1*, *sFLT1-i13*, and *sFLT1-e15a* were significantly up-regulated. In particular, the *sFLT1* mRNA expression was mainly increased (Fig. 3a). To confirm these results, an ELISA was performed on the conditioned media from vehicle- and 5azadC-treated cells. As shown in Fig. 3b, the secretion of sFLT1 into the conditioned media was observed in the 5azadC-treated cells and not in the vehicle-treated cells. The 5azadC-induced production of sFLT1 proteins was confirmed by Western blotting of the cell lysates and conditioned media from these cells. The expression of both the sFLT1-i13 and sFLT1-e15a proteins in the cell lysates was observed in all three cell lines, whereas tmFLT1 protein expression was undetectable (Fig. 3c). Furthermore, the secretion of these sFLT1 proteins into the conditioned media was detected in all three cell lines (Fig. 3d). These results indicate that sFLT1 protein expression was induced by 5azadC treatment.

### Hypermethylation of CpG sites located in the promoter region of the *FLT1* gene in choriocarcinoma cells

**Fig. 4:**
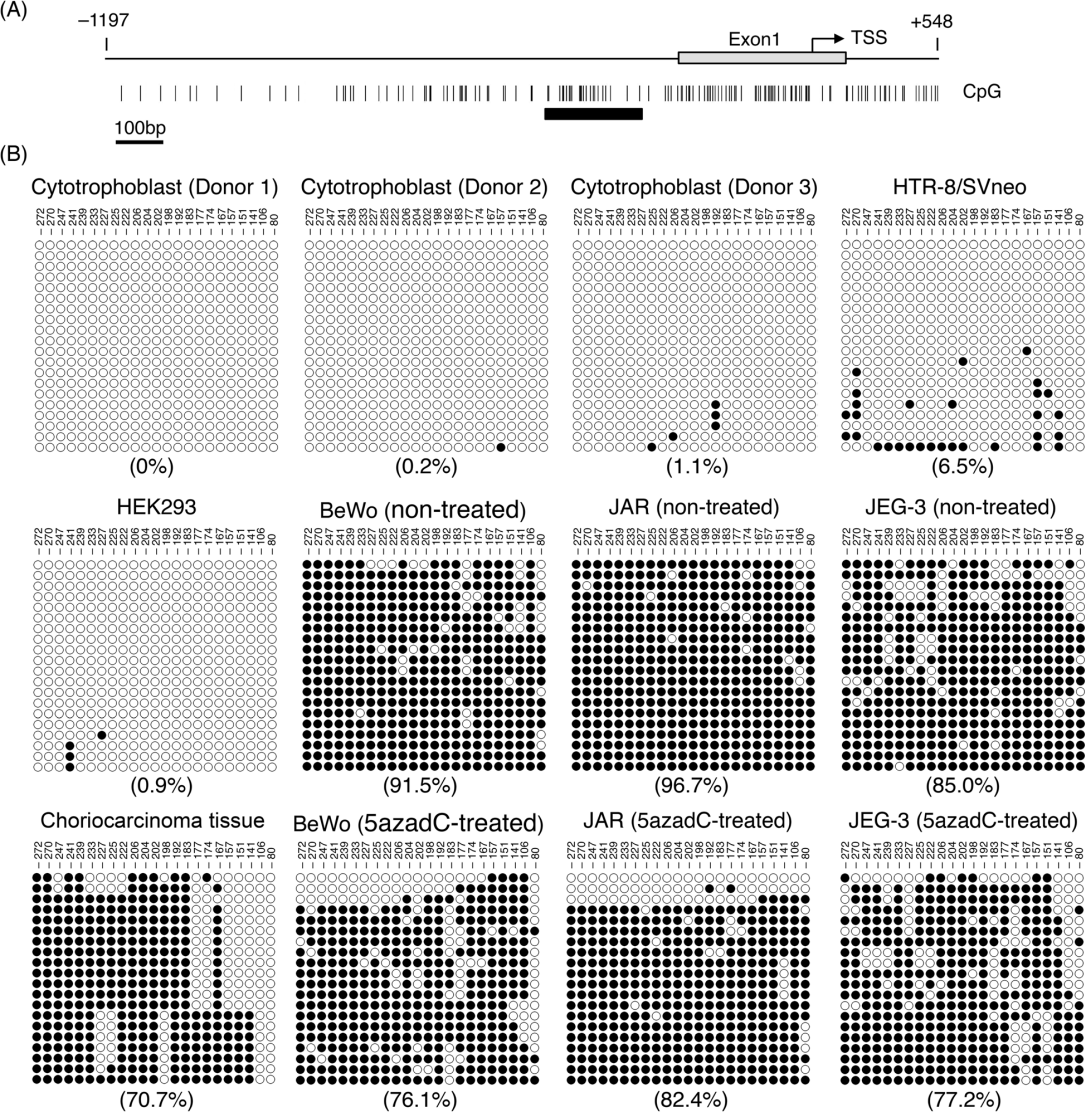
Hypermethylation of the *FLT1* promoter in cells and a tissue from choriocarcinoma. **a** Schematic diagram of the genomic structure of the *FLT1* gene. Numbers indicate the position relative to the transcription start site (TSS) of *FLT1*. The vertical lines represent the location of CpG dinucleotides. The thick bar indicates a typical CpG island in the *FLT1* promoter. **b** Methylation patterns in the promoter region of *FLT1*. The 23 CpG sites of *FLT1* were analyzed by bisulfite sequencing. The DNA methylation data were analyzed by the QUMA methylation analysis tool (http://quma.cdb.riken.jp/). Open circles and closed circles represent unmethylated and methylated CpG sites, respectively. Numbers indicate the position relative to the TSS. Numbers in parentheses represent the overall percentage of methylated CpG sites

Since sFLT1 protein expression was induced by 5azadC treatment in three choriocarcinoma cell lines, we investigated the DNA methylation pattern of the *FLT1* promoter region in cytotrophoblasts, HTR-8/SVneo cells, HEK293 cells, choriocarcinoma cells, and a tumor specimen. The methylation status of a typical CpG island in the human *FLT1* promoter was characterized using bisulfite DNA sequencing (Fig. 4a). We sequenced 20 individual DNA clones derived from each sample. In cytotrophoblasts, HTR-8/SVneo, and HEK293 cells most of the CpG sites in the CpG island of the *FLT1* promoter were not methylated (Fig. 4b). By contrast, in the BeWo, JAR, and JEG-3 cells a high degree of methylation was observed at CpG sites (Fig. 4b). Similarly, as expected, the degree of methylation was also high in a clinical specimen (Fig. 4b).

Next, the methylation status of the *FLT1* promoter region in the three choriocarcinoma cell lines after 5azadC treatment for 5 days was evaluated. 5azadC treatment reduced the overall percentage of methylated CpG sites in the *FLT1* promoter region from 91.5 to 76.1% in BeWo cells, from 96.7 to 82.4% in JAR cells, and from 85.0 to 77.2% in JEG-3 cells (Fig. 4b). Meanwhile, in HTR-8/SVneo cells, which were hypomethylated in the *FLT1* promoter region, 5azadC treatment did not induce any significant up-regulation in the *total-FLT1* mRNA expression (Additional file 1: Figure S4). These findings suggest that *FLT1* gene silencing in choriocarcinoma cells is associated with the DNA methylation of its promoter region.

### Hypoxia induces increased *sFLT1* mRNA expression and elevated sFLT1 production in 5azadC-treated choriocarcinoma cells

**Fig. 5:**
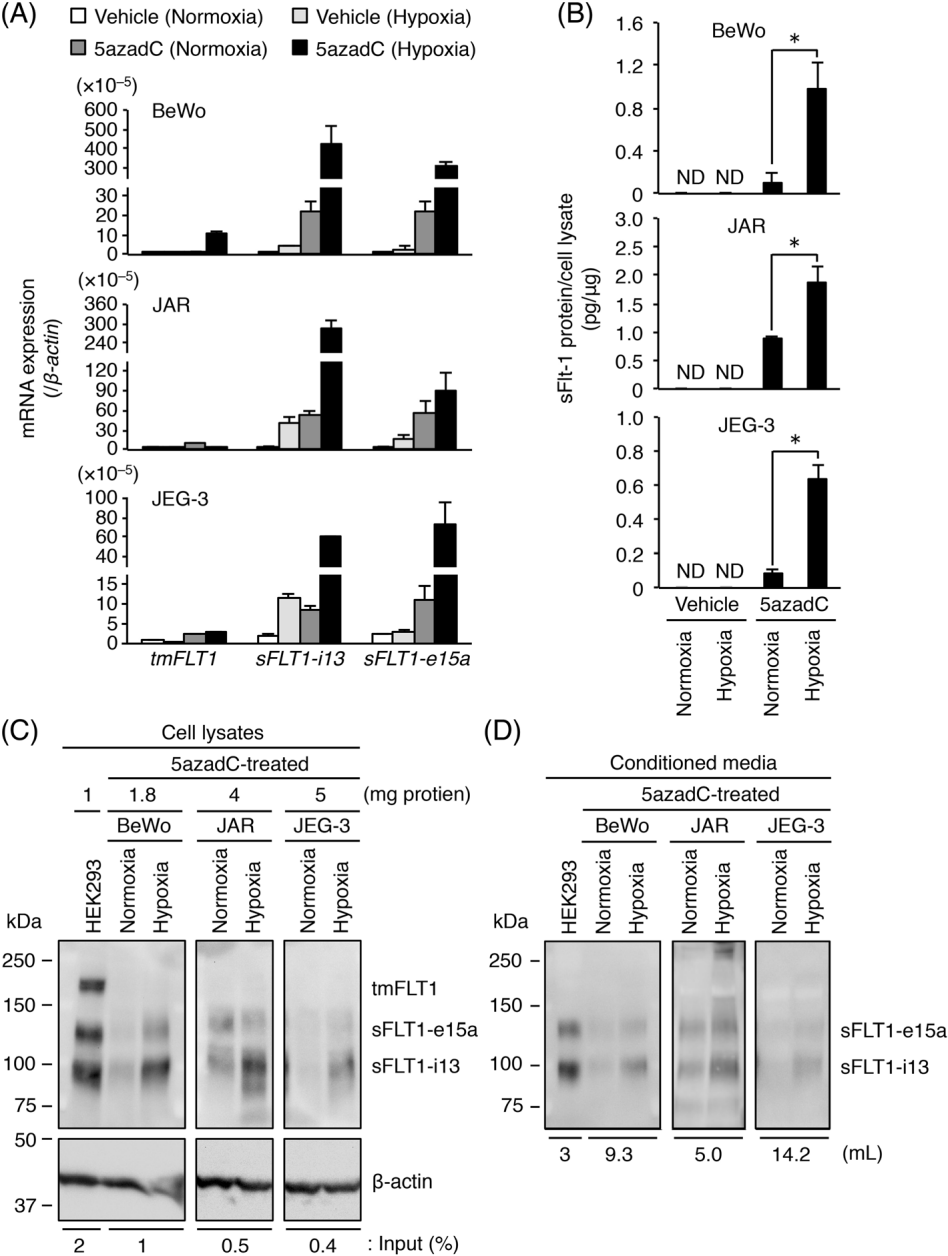
Hypoxia-induced up-regulation of sFLT1 protein secretion in 5azadC-treated choriocarcinoma cells. Three choriocarcinoma cell lines were incubated in the presence of 0.1% DMSO (vehicle control) or 10 μM 5azadC. Culture media were changed daily. After 5 days of culture, culture media were changed to fresh growth media without 5azadC and then cells were incubated for 24 h under normoxic or hypoxic conditions. Conditioned media were collected for ELISA and Western blotting. **a** The mRNA expression levels of three *FLT1* splice variants in the three choriocarcinoma cell lines. Results are expressed as a ratio relative to the expression of *β-actin* mRNA. **b** Measurement of sFLT1 secreted from the three choriocarcinoma cell lines. The amount of sFLT1 in the conditioned media was measured by ELISA, and then normalized to the protein content of each cell. **c** Immunoprecipitation of FLT1 isoforms from the cell lysates derived from the three 5azadC-treated cell lines under normoxic or hypoxic conditions. Numbers represent the amount of protein subjected to immunoprecipitation. The indicated percentage of cell lysate was used as the input. **d** Western blotting of sFLT1 proteins secreted into the conditioned media. Numbers indicate the volume of media subjected to heparin-affinity pull-down. Uncropped images of Western blots are presented in Additional file 1: Figure S6. All values represent the mean ± SD (n = 3). Asterisks indicate a significant difference (P < 0.05). ND: not detected

Recently, we reported that hypoxia-inducible factor-2α (HIF-2α) mediates the hypoxia-induced up-regulation of *FLT1* gene expression in the BeWo, JAR and JEG-3 three choriocarcinoma cell lines [21]. Under hypoxia, HIF-2α forms a heterodimer with HIF-1β, binds to the hypoxia response element (HRE) of target genes, and induces hypoxia-related gene expression [24]. Because the HRE motif 5′-(A/G)CGTG-3′ also contains one CpG dinucleotide, we investigated the effects of hypoxic stimulation on *FLT1* expression and sFLT1 production in the three choriocarcinoma cell lines treated with 5azadC. All cells were treated with 10 μM 5azadC for 5 days prior to exposure to either normoxia or hypoxia for 24 h. As shown in Fig. 5a, under hypoxic conditions the mRNA expression of both *sFLT1-i13* and *sFLT1-e15a* was up-regulated in the 5azadC-treated cells compared with that in the vehicle-treated cells. These results were confirmed at the protein level by performing an ELISA on the conditioned media. A significant hypoxia-induced increase in sFLT1 production was observed in the 5azadC-treated cells, whereas the protein expression of sFLT1 was not detected in vehicle-treated cells under normoxic or hypoxic conditions (Fig. 5b). Moreover, the protein expression of sFLT1 isoforms in the cell lysates and conditioned media from 5azadC-treated cells was confirmed by Western blotting. The protein expression and secretion of both sFLT1-i13 and sFLT1-e15a were elevated by hypoxic stimulation (Fig. 5c, d). These results suggest that HRE motif(s) in the *FLT1* gene are methylated in choriocarcinoma cells, resulting in the suppression of hypoxia-induced sFLT1 production.

### Inhibition of in vivo tumorigenicity in choriocarcinoma cells by stable expression of sFLT1

In the three choriocarcinoma cell lines, we found that *FLT1* gene expression was suppressed by DNA methylation of the gene promoter region. The three cell lines are known to develop tumors in nude mice [4]. In the JAR and JEG-3 xenograft models, administration of sFLT1 chimeric protein has been shown to significantly inhibit their tumor growth [4]. In addition, it has been reported that *sFLT1* gene transfer is potent in inhibiting tumor growth using various tumor graft models [25–28], and bioactivities of both sFLT1-i13 and sFLT1-e15a are nearly comparable [29]. Therefore, while similar results may be obtained as described above, the in vivo tumorigenicity when sFLT1-i13 over-expressed in JEG-3 cells with the highest tumor growth rate among the three cell lines was investigated. The stable sFLT1-i13- or GFP-expressing JEG-3 cells were established and designated as JEG3-i13 and JEG3-GFP, respectively. We confirmed that over-expressed sFLT1-i13 did not affect the cell proliferation of JEG-3 cells in vitro (Additional file 1: Figure S5).

**Fig. 6:**
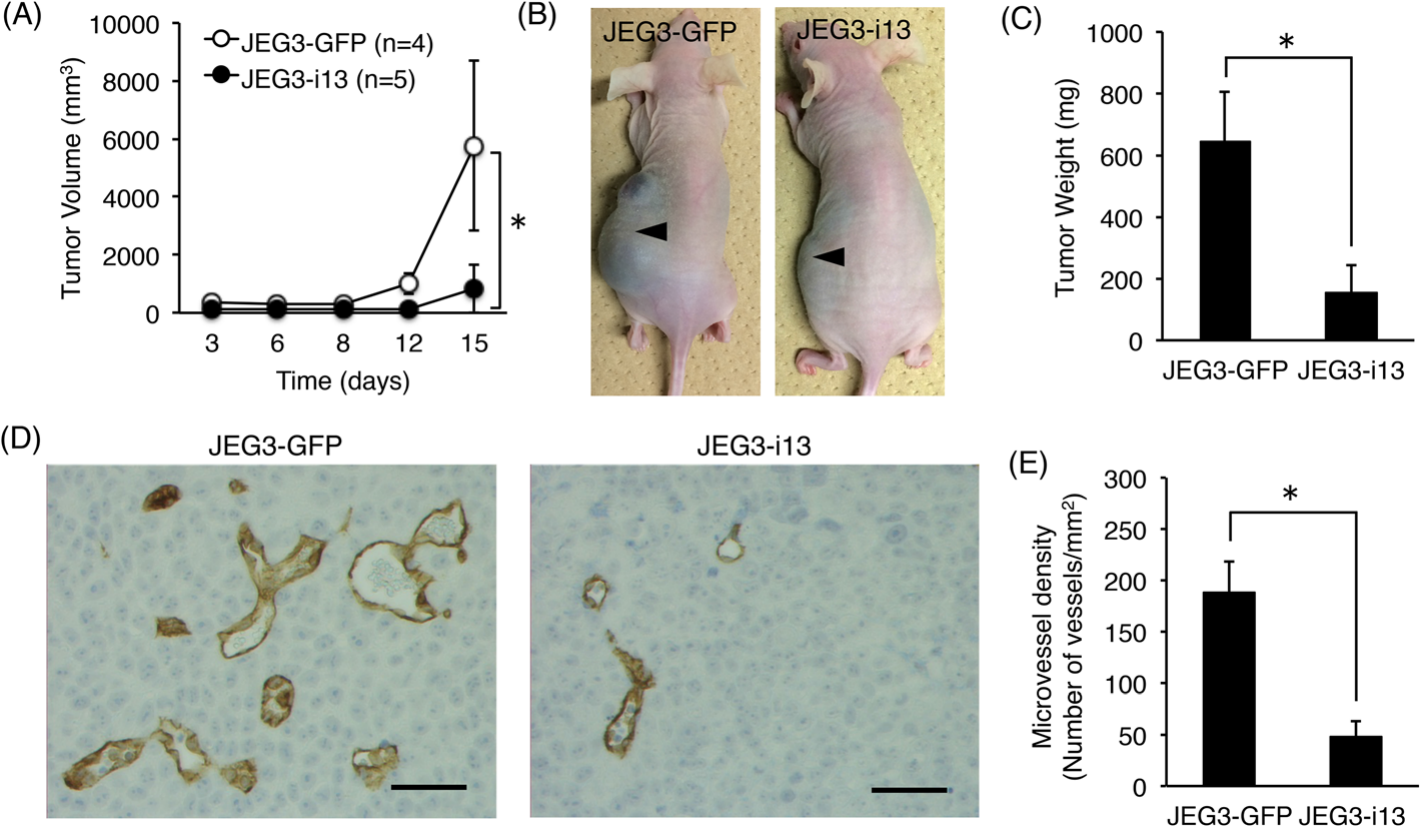
sFLT1 expression in choriocarcinoma cells significantly reduces in vivo tumorigenic activity. The stable sFLT1-i13- or GFP-expressing JEG-3 cells were designated as JEG3-i13 and JEG3-GFP, respectively. **a** Quantitation of tumor size in JEG3-i13- or JEG3-GFP-implanted groups. **b** A significant decrease in the size of tumor nodules derived from JEG3-i13 cells at day 15 after subcutaneous injection into nude mice. Arrowheads indicate the tumors grown at injection sites. **c** The mean weight of tumors. **d** Immunohistochemical analysis of microvessel formation in tumors. The microvessels were shown as a brown color when stained using anti-mouse CD31 monoclonal antibody. **e** Quantitative analysis of microvessel density in tumors. Scale bar: 50 μm. All values represent the mean ± SD. Asterisks indicate a significant difference (*P* < 0.05)

Next, JEG3-i13 (*n* = 5) and JEG3-GFP (*n* = 4) cells were subcutaneously implanted into nude mice. As shown in Fig. 6a-c, the volume, size, and mean weight of tumors were significantly lower in mice transplanted with JEG3-i13 cells than the JEG3-GFP control cells. Moreover, the number of microvessels in mice implanted with JEG3-i13 cells was significantly lower than that in mice implanted with JEG3-GFP cells (Fig. 6d, e). These results demonstrate that the stable expression of sFLT1 in choriocarcinoma cells resulted in suppressed tumor growth and microvessel formation in vivo.

## Discussion

In this study, we found that the strong *FLT1* suppression observed in choriocarcinoma cells is caused by DNA hypermethylation of its 5′-promoter region, and showed that DNA demethylation by 5azadC rescued sFLT1 production. Furthermore, we demonstrated that the stable expression of sFLT1 in choriocarcinoma cells resulted in the suppression of tumor growth and tumor vascularization in vivo. These results suggest that *FLT1* may be a tumor suppressor gene in choriocarcinoma.

Promoter hypermethylation is known to play a key role in the epigenetic silencing of tumor suppressor genes in the development and progression of cancers [17]. In this study, we showed that the *FLT1* promoter was hypermethylated in all three choriocarcinoma cell lines. The methylation status of *FLT1* has also been reported in other cancer cell lines and tissue samples besides choriocarcinoma. For example, Yamada et al. reported that the promoter and exon 1 of *FLT1* were aberrantly methylated in prostate cancer cell lines and tumor tissue samples, whereas benign prostate tissue samples were hypomethylated [30]. Furthermore, Kim et al. reported that the *FLT1* promoter showed variable hypermethylation in different cancer cell lines, including colon, stomach, lung, melanocyte, breast, thyroid, and kidney [31, 32]. Among those, the colon, stomach and renal cancer cell lines had more frequent *FLT1* hypermethylation than the other cancer cell lines. Methylation of the *FLT1* promoter was also found to be significantly higher in the tumor tissues of stomach cancer, colon cancer, hepatocellular carcinoma, and renal cancer, in comparison with normal tissues. Therefore, the methylation status of *FLT1* promoter may be useful as a new potential biomarker for cancer diagnosis.

It is widely accepted that HIF-α is stabilized at protein level, then translocates to the nucleus where it forms a heterodimer with HIF-1β under hypoxic or hypoxia-mimicking conditions, inducing the expression of hypoxia-associated genes. During this process, the HIF-α/HIF-1β heterodimer binds the consensus HRE sequence 5′-(A/G)CGTG-3′ of the target genes [24]. We recently discovered that HIF-2α, not HIF-1α, mediates the hypoxia-induced up-regulation of *FLT1* expression in choriocarcinoma cell lines (BeWo, JAR, and JEG-3) and primary trophoblasts [21]. The HRE motif contains one CpG dinucleotide, and it has been confirmed that CpG methylation within this sequence prevents the binding of HIF-1α or HIF-2α and subsequently prevents the HIF-mediated transcription of HRE-containing genes [33]. Our results suggest that HRE motifs in *FLT1* were methylated, suppressing the binding of HIF-α in all three choriocarcinoma cell lines, as sFLT1 production was enhanced by hypoxic stimulation after 5azadC treatment. A putative HRE motif was reported to exist approximately 1000 bp upstream of the transcription start site in mouse and human *FLT1* genes [34]. The next step is to investigate whether HIF-2α binds to the HRE site when exposed under hypoxic conditions and to assess the CpG methylation status of the HRE site before and after 5azadC treatment.

Previously we reported that both the CRE (cyclic adenosine monophosphate response element) and ETS (E26 transformation-specific) motifs located in the region 90 bp upstream of the transcription start site are important for *FLT1* promoter activity in human embryonic kidney 293E1 cells [35]. However, the transcription factors binding with each motif have not been fully determined. The CRE motif 5′-TGACGTCA-3′ also contains one CpG dinucleotide, and CpG methylation of the CRE motif has been reported to inhibit the binding of specific transcription factors and transcriptional activation [36]. In the BeWo, JAR, and JEG-3 cells, DNA methylation was also observed at a CpG dinucleotide located in the CRE motif (position − 81 of the *FLT1* promoter), and its methylation level was decreased in all these cells compared to before 5azadC treatment (Fig. 4b). By contrast, the CpG dinucleotide in the CRE motif was unmethylated in the human primary choriocarcinoma tissue specimen (Fig. 4b). Further studies are required to confirm whether the CRE motif at this site is important for *FLT1* promoter activation in human choriocarcinoma cells.

First-line treatment of choriocarcinoma is well-established as multiagent chemotherapy using etoposide, methotrexate, actinomycin D, cyclophosphamide, and vincristine (abbreviated as EMA/CO). In addition, other treatments including EP/EMA (etoposide, cisplatin, methotrexate, and actinomycin D), TP/TE (paclitaxel, cisplatin, and etoposide), BEP (bleomycin, etoposide, and cisplatin), ICE (ifosfamide, carboplatin, and etoposide) and FA (5-fluorouracil and actinomycin D) are also effective [37]. However, the systemic administration of anticancer drugs sometimes results in nonselective drug distribution and severe side effects such as bleeding and respiratory failure. In this study, stable expression of sFLT1 in choriocarcinoma cells resulted in strong suppression of tumor growth and vascularity in vivo. Therefore, the over-expression of sFLT1 using a DNA demethylating agent could be a novel therapeutic alternative to anticancer drugs in choriocarcinoma. In this study the 5azadC, also known as decitabine, was used as a potent DNA methylation inhibitor [38]. This agent is also used as a drug for the treatment of myelogenous leukemia and solid tumors in lung cancer, esophageal cancer, and pleural mesothelioma [38, 39], however its efficacy is limited by the 35 min half-life in plasma of humans [38]. To address this issue, guadecitabine, a novel hypomethylating dinucleotide of decitabine and deoxyguanosine, has been developed [40]. This demethylating agent gradually releases the active metabolite decitabine because it is resistant to degradation by cytidine deaminase. Albany et al. have recently reported that guadecitabine administration induces regression of xenografts in a mouse model of human germ cell tumors [41]. Furthermore, it has been reported that the drug delivery systems using nanogels or nanoparticles can also overcome this limitation and enhance chemotherapeutic efficacy [42, 43]. Thus, these choriocarcinoma therapies targeting methylated DNAs could be offered in the future.

## Conclusion

This study demonstrated that the inhibition of sFLT1 production by *FLT1* gene silencing occurs via the hypermethylation of its promoter in choriocarcinoma cells. These findings contribute to our understanding of the mechanism of choriocarcinogenesis, and suggest a novel molecular targeting therapy for the treatment of choriocarcinoma.

## Supplementary information

**Additional file 1: Figure S1.** HEK293 cells express three *FLT1* splice variants and secrete two sFLT1 isoforms. **Figure S2.** The mRNA expression level of all *FLT1* transcript variants (*total-FLT1*) in a human primary choriocarcinoma tissue specimen. **Figure S3.** Effect of 5-aza-2′-deoxycytidine (5azadC) on the *FLT1* gene expression in choriocarcinoma cells. **Figure S4.** Effect of 5azadC on the *FLT1* expression in HTR-8/SVneo cells. **Figure S5.** Characterization of stable sFLT1-i13- or GFP-expressing JEG-3 cells. **Figure S6.** Uncropped images of Western blots shown in the main and supplementary figures. Table S1. Oligonucleotide primer sequences for qRT-PCR.

**Additional file 2.** Supplementary methods of siRNA transfection in Figure S1 and cell proliferation assay in Figure S5.

## Data Availability

All data generated or analyzed during this study are included in this published article and its supplementary information files.

## Abbreviations

5azadC: 5-aza-2′-deoxycytidine
sFLT1: Soluble fms-like tyrosine kinase-1
VEGF: Vascular endothelial growth factor
