# Supplementary material for: Production of an anti-angiogenic factor sFLT1 is suppressed via promoter hypermethylation of FLT1 gene in choriocarcinoma cells
Source: BMC Cancer. 2020 Feb 10;20:112. doi: 10.1186/s12885-020-6598-9 (PMC7011436; doi:10.1186/s12885-020-6598-9)
Supplement: Supplementary file 1 — Additional file 1: Figure S1. HEK293 cells express three FLT1 splice variants and secrete two sFLT1 isoforms. Figure S2. The mRNA expression level of all FLT1 transcript variants (total-FLT1) in a human primary choriocarcinoma tissue specimen. Figure S3. Effect of 5-aza-2′-deoxycytidine (5azadC) on the FLT1 gene expression in choriocarcinoma cells. Figure S4. Effect of 5azadC on the FLT1 expression in HTR-8/SVneo cells. Figure S5. Characterization of stable sFLT1-i13- or GFP-expressing JEG-3 cells. Figure S6. Uncropped images of Western blots shown in the main and supplementary figures. Table S1. Oligonucleotide primer sequences for qRT-PCR. [file 12885_2020_6598_MOESM1_ESM.pdf]

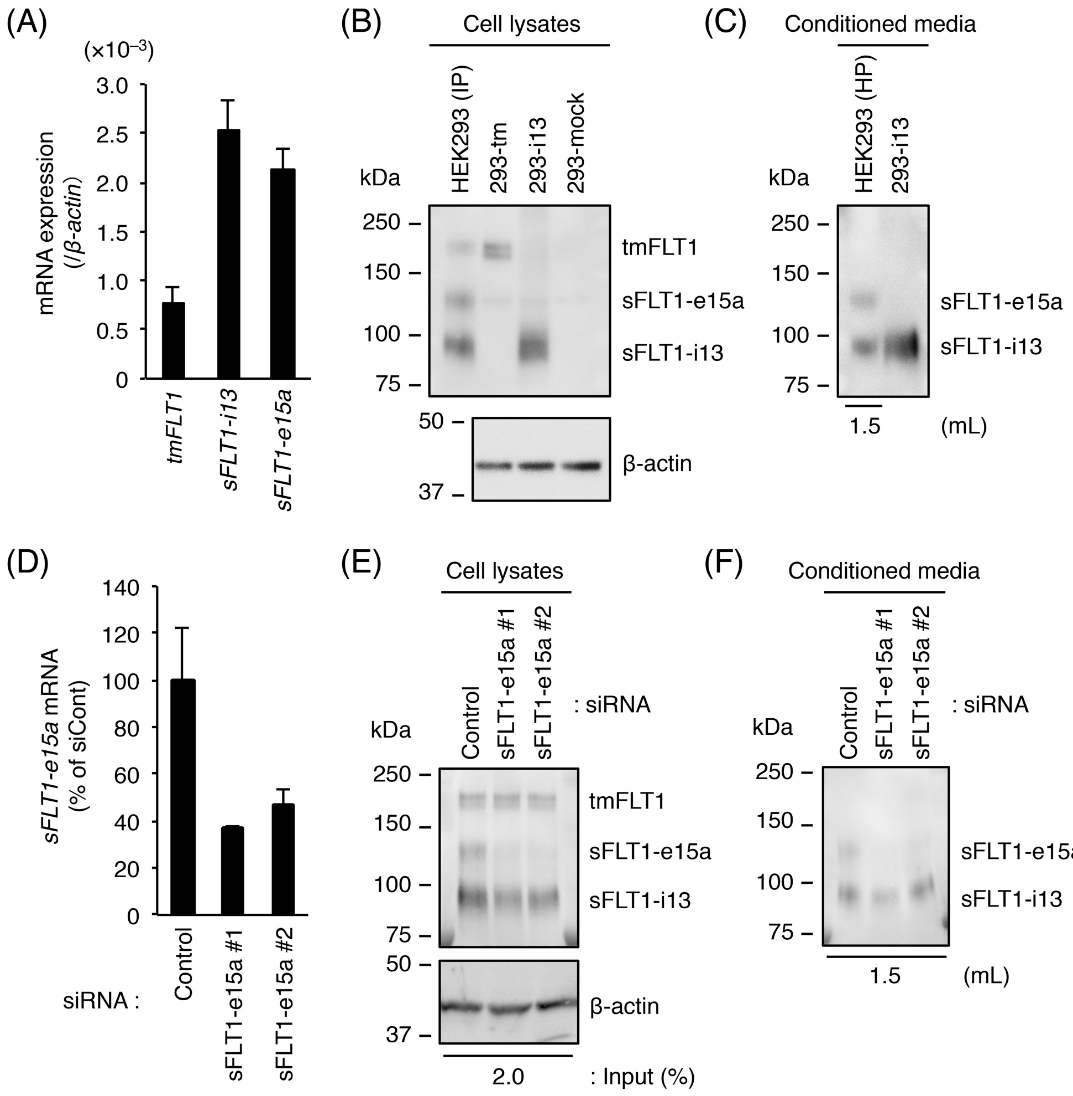

Figure S1 HEK293 cells express three *FLT1* splice variants and secrete two sFLT1 isoforms. (A) The mRNA expression level of three *FLT1* splice variants (*tmFLT1, sFLT1-i13, and sFLT1-e15a*) in HEK293 cells. (B) Western blot analysis of FLT1 isoforms in parental HEK293 cells and stable transfectants expressing tmFLT1 or sFLT1-i13. Cell lysates (1 mg protein) from parental HEK293 cells were subjected to immunoprecipitation (IP) with anti-human FLT1 monoclonal antibody. The empty vector-transfected HEK293 cells, tmFLT1-expressing cells, and sFLT1-i13-expressiong cells were designated as 293-mock, 293-tm, and 293-i13, respectively. (C) Western blot analysis of the secreted sFLT1 proteins. Conditioned media (1.5 mL) from parental HEK293 cells were subjected to heparin-affinity pull-down (HP) to concentrate sFLT1 proteins. (D) Evaluation of *sFLT1-e15a* mRNA knockdown by siRNA transfection. The siRNA transfection method is described in details in Additional file 2. (E) Immunoprecipitation of FLT1 isoforms in the cell lysates of HEK293 cells transfected with siRNA targeting *sFLT1-e15a*. Two percent of the cell lysates was used as the input. (F) sFLT1 proteins secreted in the conditioned media from HEK293 cells transfected with siRNA for *sFLT1-e15a* or control. Uncropped images of Western blots are presented in Additional file 1: Figure S6. All values represent the mean ± SD (n = 3).

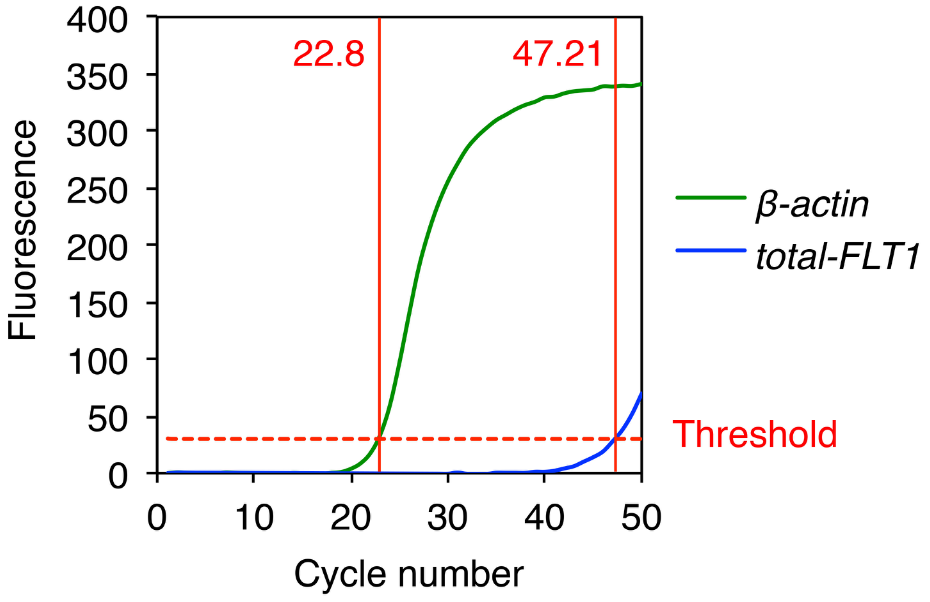

Figure S2 The mRNA expression level of all *FLT1* transcript variants (*total-FLT1*) in a human primary choriocarcinoma tissue specimen.

The mRNA level of *total-FLT1* in a primary choriocarcinoma tissue was measured by qRT-PCR using *β-actin* mRNA as a reference, and was approximately 200-fold lower than that in BeWo cells shown in Fig. 2A.

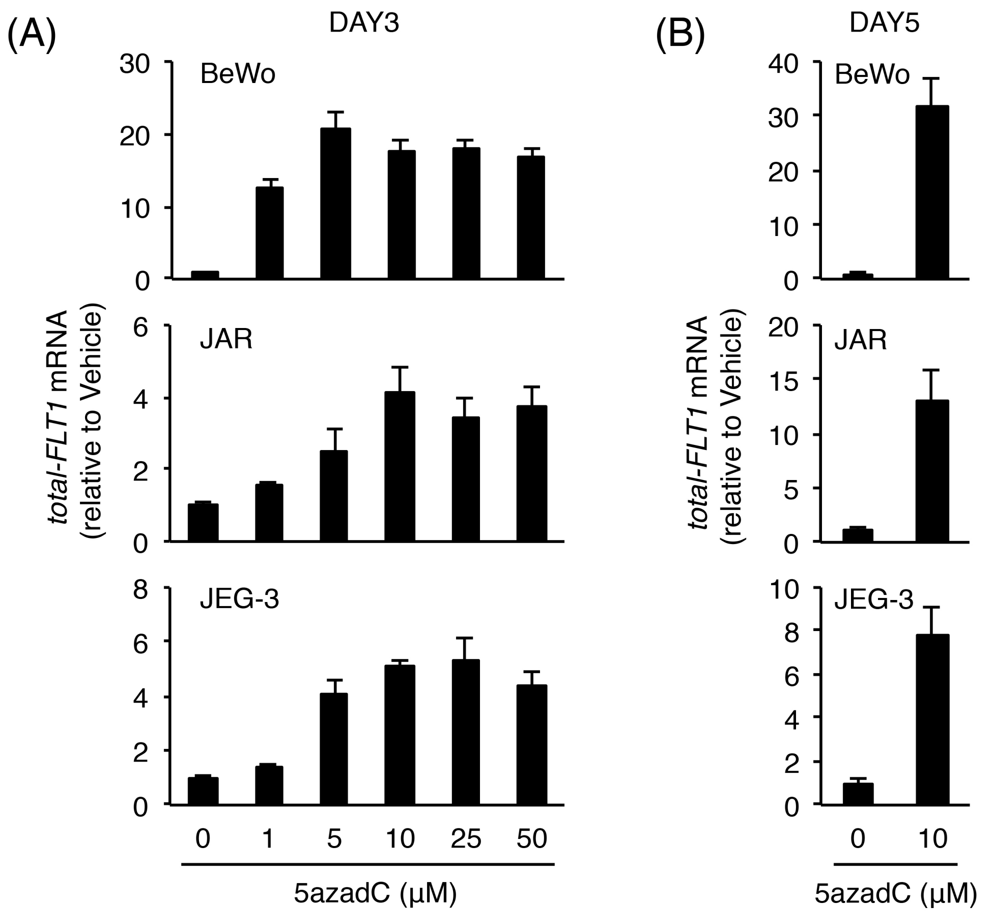

Figure S3 Effects of 5-aza-2'-deoxycytidine (5azadC) on the *FLT1* gene expression in choriocarcinoma cells.

(A) Three choriocarcinoma cell lines, BeWo, JAR, and JEG-3, were treated with various doses of 5azadC for 3 days. The mRNA expression levels of all *FLT1* transcript variants (*total-FLT1*) were measured by qRT-PCR using *GAPDH* mRNA as a reference. Results are expressed as a fold change relative to the vehicle-treated cells. (B) The mRNA expression levels of *total-FLT1* in the three choriocarcinoma cell lines treated with 10μM 5azadC for 5 days. All values represent the mean ± SD (n = 3).

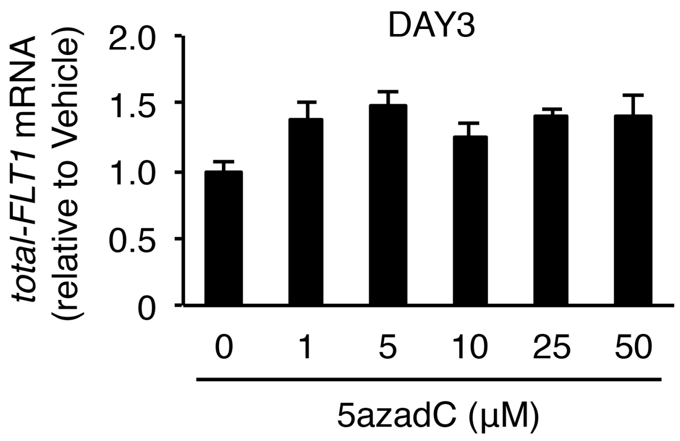

Figure S4 Effects of 5azadC on the *FLT1* expression in HTR-8/SVneo cells.

HTR-8/SVneo cells were treated with varying doses of 5azadC for 3 days. The mRNA expression levels of all *FLT1* transcript variants (*total-FLT1*) were determined by qRT-PCR analysis using *GAPDH* mRNA as a reference. Results are expressed as a fold change relative to the vehicle-treated cells. All values represent the mean ± SD (n = 3).

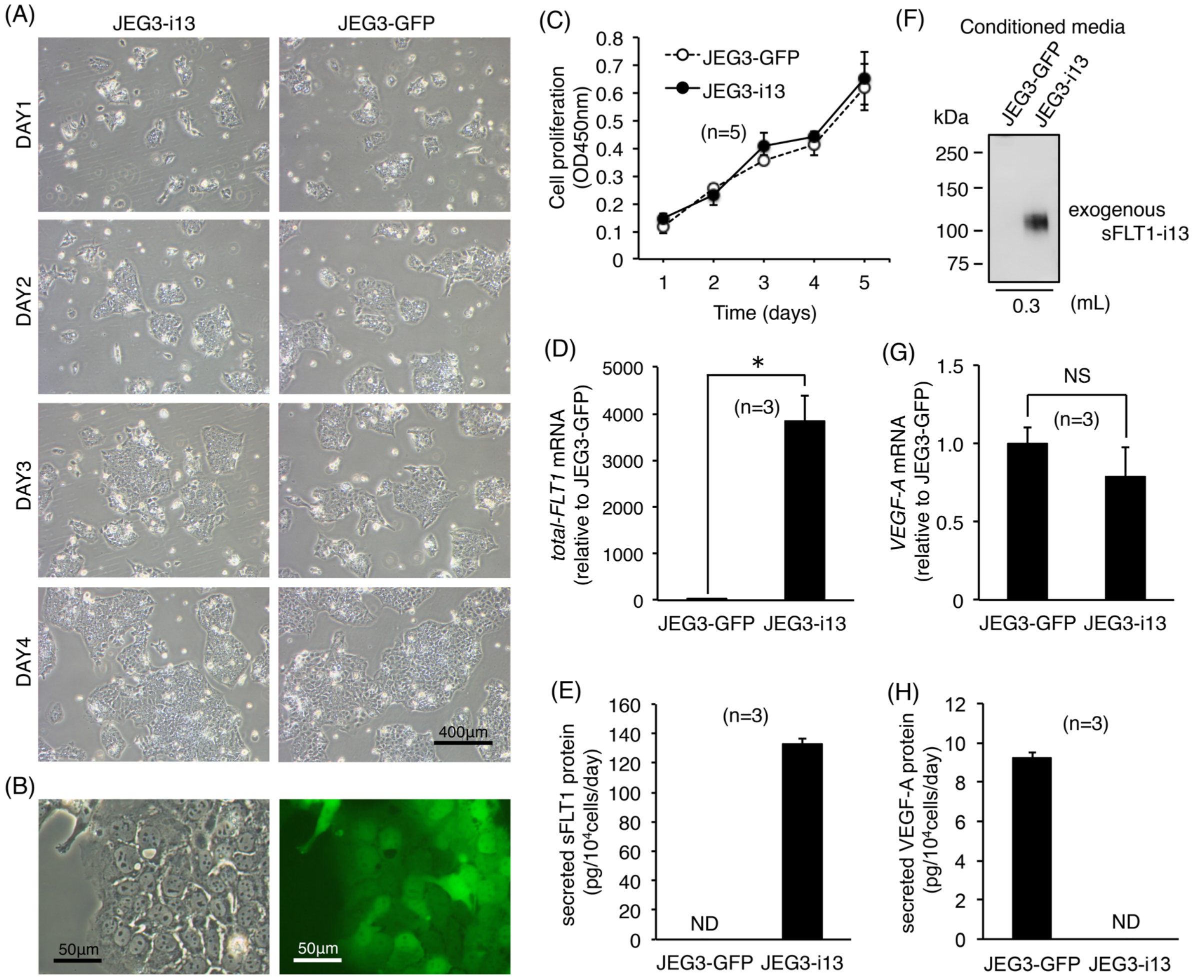

Figure S5 Characterization of stable sFLT1-i13- or GFP-expressing JEG-3 cells.

The stable sFLT1-i13- or GFP-expressing JEG-3 cells were designated as JEG3-i13 and JEG3-GFP, respectively. (A) Morphological changes. (B) Confirmation of the forced expression of GFP in JEG3-GFP cells. (C) The growth rate of JEG3-i13 and JEG3-GFP cells *in vitro* was similar. (D) The mRNA expression levels of all *FLT1* transcript variants (*total-FLT1*) in JEG-3 cells expressing sFLT1-i13 or GFP. (E) The amount of sFLT1 in the conditioned media cultured for 1 day was measured by ELISA, and then normalized to the number of cells in each cell line. (F) Secretion of sFLT1 from JEG3-i13 cells. Numbers show the volume of media subjected to heparin-affinity pull-down. Uncropped images of Western blots are presented in Additional file 1: Figure S6. (G) The mRNA expression levels of *VEGF-A* in JEG-3 cells expressing sFLT1-i13 or GFP. (H) The amount of VEGF-A in the conditioned media cultured for 1 day was measured by ELISA, and then normalized to the number of cells in each cell line. The secreted VEGF-A was detected only in the JEG3-GFP cells. It could be that the concentration of VEGF-A secreted from JEG3-i13 cells was not measured by the ELISA kit used in this study because VEGF-A was sequestered by the sFLT1-i13 expressed. ND: not detected. All values represent the mean ± SD. Asterisks indicate a significant difference ($P < 0.05$). NS: No significance.

Figure 2 (C) : left | Figure 2 (C) : middle | Figure 2 (C) : right

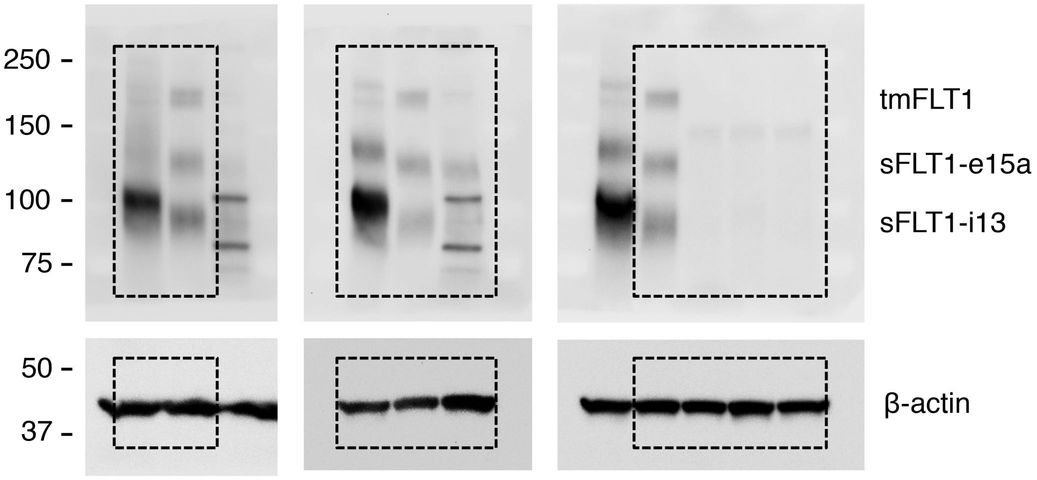

Figure 2 (D)

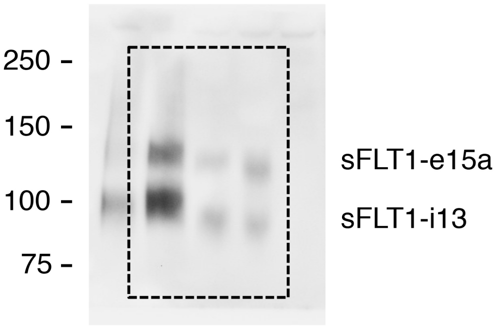

Figure 3 (C) : left | Figure 3 (C) : middle | Figure 3 (C) : right

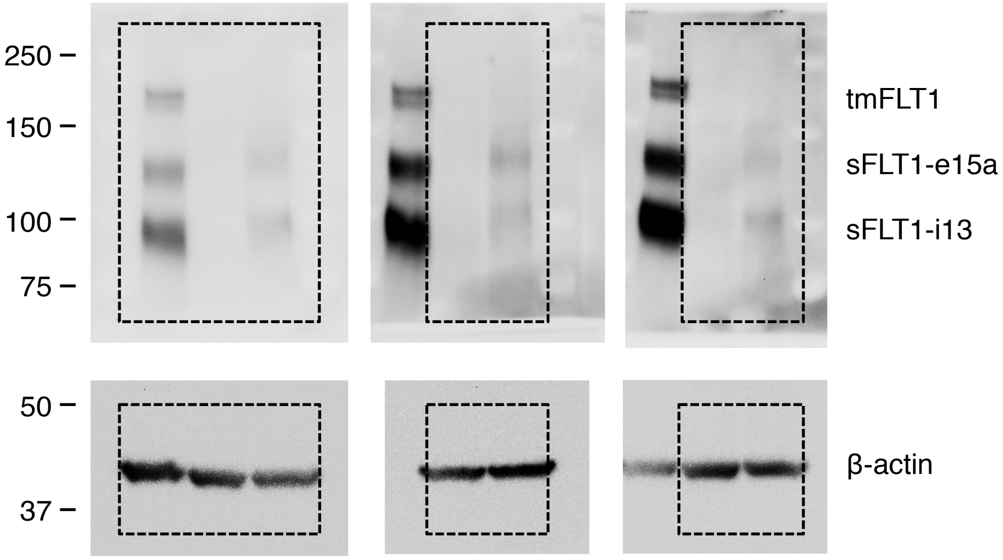

Figure 3 (D) : left | Figure 3 (D) : middle | Figure 3 (D) : right

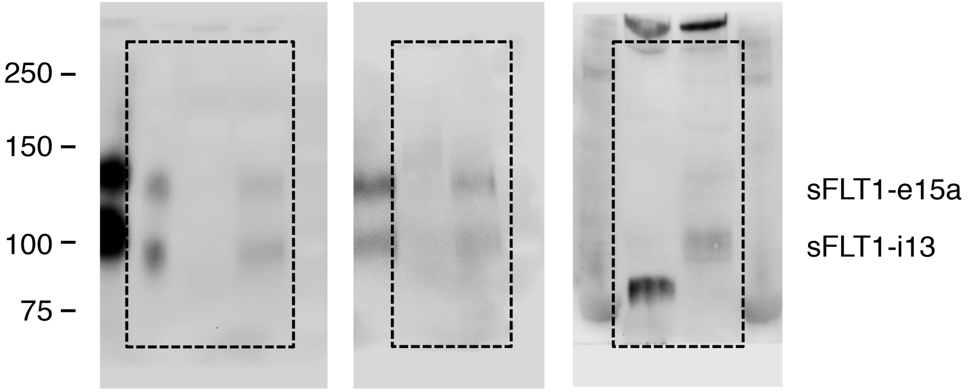

Figure 5 (C) : left | Figure 5 (C) : middle | Figure 5 (C) : right

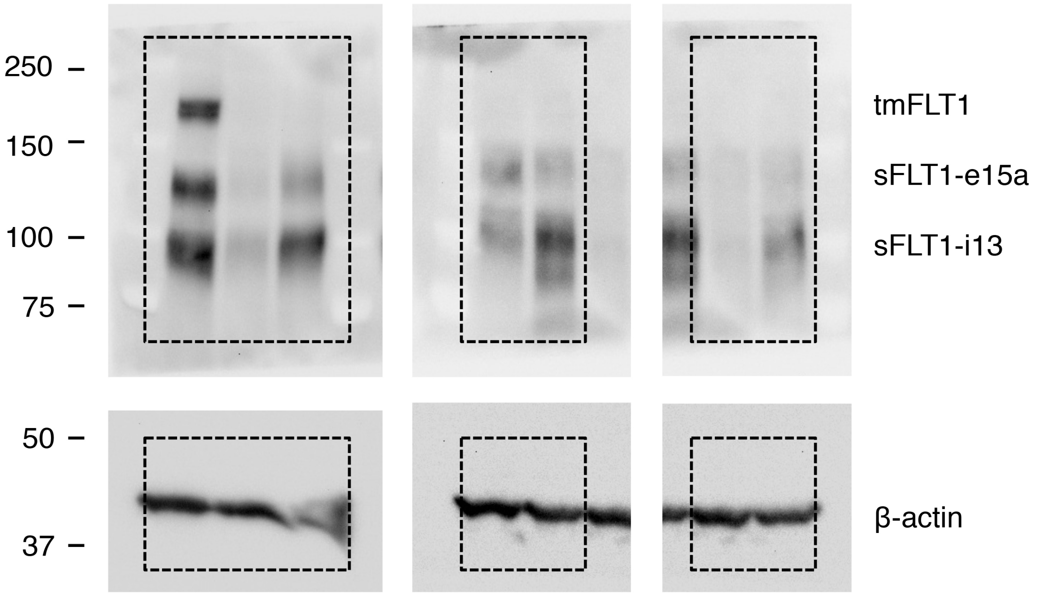

Figure 5 (D) : left | Figure 5 (D) : middle | Figure 5 (D) : right

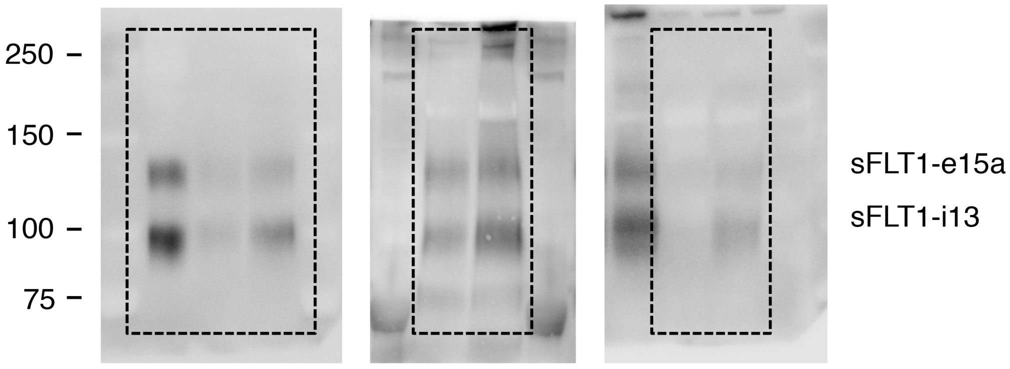

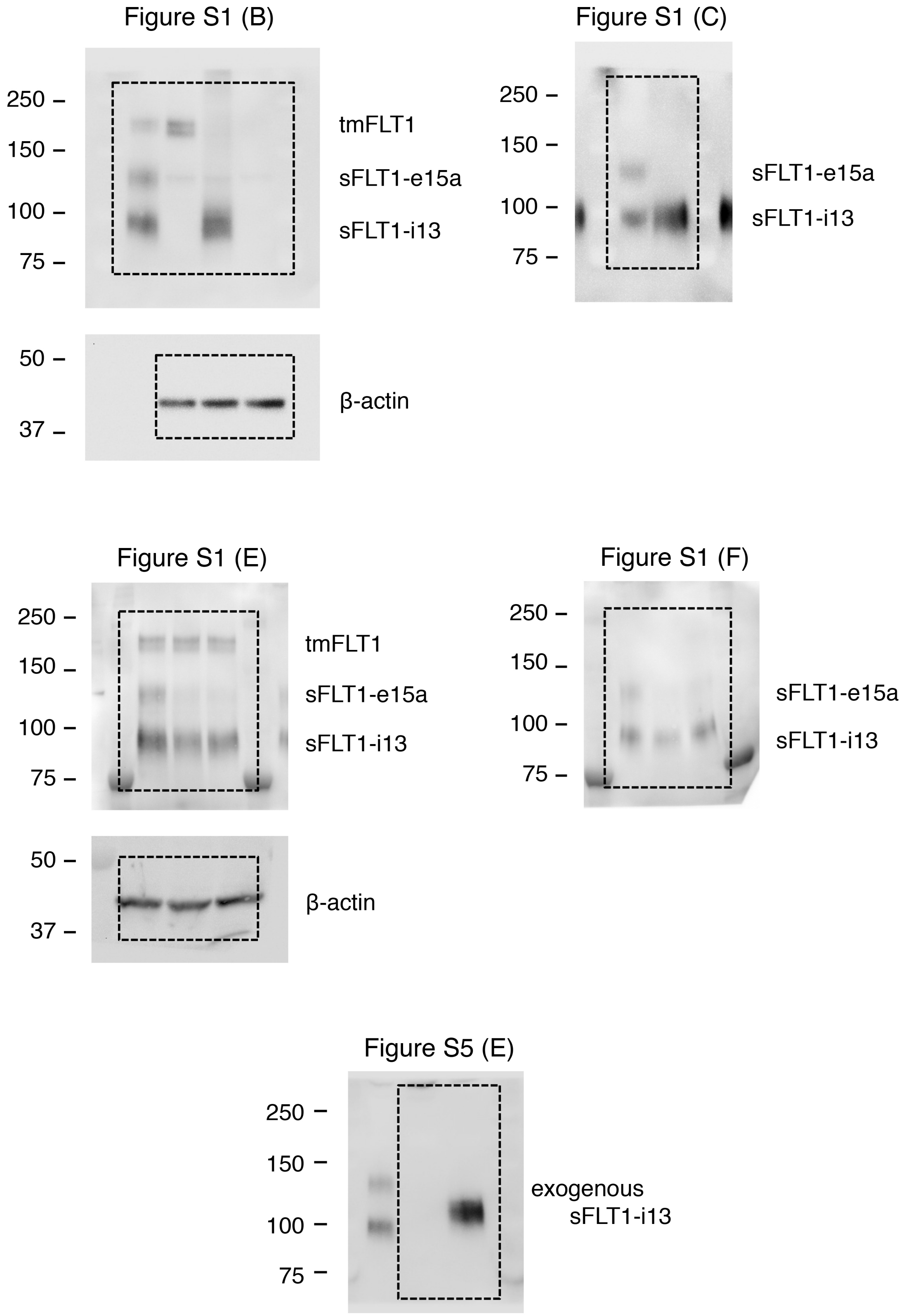

Figure S6 Uncropped images of Western blots shown in the main and supplementary figures.

Boxed areas indicate the cropped regions.

Table S1. Oligonucleotide primer sequences for qRT-PCR

Gene		Primer sequence
VEGF-A	Forward	5’-AGGAGGAGGGCAGAATCATCA-3’
	Reverse	5’-CTCGATTGGATGGCAGTAGCT-3’
total-FLT1	Forward	5’-CCCTGTAACCATAATCATTCCGAAG-3’
	Reverse	5’-TCAGCCACAACCAAGGTGCTA-3’
tmFLT1	Forward	5’-AGAGATGGGACCGTCATCAG-3’
	Reverse	5’-CTGGCTCTAGCCTGCTTTTG-3’
sFLT1-i13	Forward	5’-ACTTGGTGCACGTTTGGATT-3’
	Reverse	5’-AGAGGTTGGCATCAAAATGG-3’
sFLT1-e15a	Forward	5’-AGTTGGAGAGCCAAGACAATC-3’
	Reverse	5’-CAGCATTTCACCATCTTGGTC -3’
β-actin	Forward	5’-AAATCTGGCACCACACCTTC-3’
	Reverse	5’-TGATCTGGGTCATCTTCTCG -3’
GAPDH	Forward	5’-CAATGACCCCTTCATTGACC-3’
	Reverse	5’-ATGACAAGCTTCCCGTTCTC -3’
