# Supplementary material for: Production of an anti-angiogenic factor sFLT1 is suppressed via promoter hypermethylation of FLT1 gene in choriocarcinoma cells
Source: BMC Cancer. 2020 Feb 10;20:112. doi: 10.1186/s12885-020-6598-9 (PMC7011436; doi:10.1186/s12885-020-6598-9)
Supplement: Supplementary file 2 — Additional file 2. Supplementary methods of siRNA transfection in Figure S1 and cell proliferation assay in Figure S5. [file 12885_2020_6598_MOESM2_ESM.pdf]

Supplementary methods of siRNA transfection in Figure S1 and cell proliferation assay in Figure S5.

siRNA transfection

Mission siRNA Universal Negative Control #1, siRNA duplex targeting sFLT1-e15a #1 (sense 5'-GAACUGUAUACAUCAACGUtt-3', antisense 5'-ACGUUGAUGUAUACAGUUCtt-3') and siRNA duplex targeting sFLT1-e15a #2 (sense 5'-CCAUUUAUUGAAAACUAUUtt-3', antisense 5'-AAUAGUUUUCAAUAAAUGGtt-3') were synthesized by Sigma Genosys (Hokkaido, Japan). HEK293 were transfected with siRNA (30 nM) for 24 h using Lipofectamine RNAiMAX transfection reagent (Invitrogen) according to the manufacturer's instructions. After transfection, the medium was replaced with fresh growth media, and the cells were incubated for an additional 48 h. RNA extraction, protein preparation, qRT-PCR, heparin-affinity pull-down, and Western blot analysis were performed.

Cell proliferation assay

Cell proliferation was assessed using the Cell Counting Kit-8 (CCK-8) (Dojindo, Kumamoto, Japan) according to the manufacturer's instructions. Briefly, JEG3-i13 and JEG3-GFP cells were seeded on 96-well cell culture plates (Sumitomo Bakelite Co., Ltd.) at a density of 3,000 cells/well. At various time points, cells were incubated with CCK-8 reagent for 1 h at 37 ºC, then the absorbance was measured at 450 nm using a DigiScan 340T microplate reader (Asys Hitech GMBH). All experiments were carried out in quintuplicate.
